# Effect of therapeutic plasma exchange on plasma levels and total removal of adipokines and inflammatory markers

**DOI:** 10.1186/s40608-015-0067-z

**Published:** 2015-09-30

**Authors:** Julius J. Schmidt, Janine Jahn, Paulina Golla, Carsten Hafer, Jan T. Kielstein, Heike Kielstein

**Affiliations:** Department of Internal Medicine, Division of Nephrology and Hypertension, Medical School Hannover, Carl-Neuberg-Strasse 1, 30625 Hannover, Germany; Department of Anatomy and Cell Biology, Faculty of Medicine, Martin Luther University Halle-Wittenberg, Halle, Germany

## Abstract

**Background:**

Aside from well-established inflammatory mediators adipokines have recently been found to play an important role in a variety of immunologic diseases. Therapeutic plasma exchange (TPE) is an established treatment modality for the acute removal of pathophysiological relevant disease mediators. The aim of this study was to determine adipokine removal during TPE therapy.

**Methods:**

21 Caucasian patients (10 females, 11 males) with an indication for TPE using albumin as exchange fluid received two consecutive TPE sessions. Blood samples for measurement of resistin, leptin, sICAM-1, sCD40L, MCP-1, and sTNF-R were drawn before and at the end of each TPE session. Samples from the total removed plasma were collected at the end of every treatment.

**Results:**

We found a significant reduction in pre- vs. post-TPE plasma concentrations for sICAM-1 (517 ± 246 vs. 260 ± 159 ng/ml, *p* < 0.0001), sTNF-R (8.1 ± 6.4 vs. 5.7 ± 3.9 ng/ml, *p* < 0.05), and resistin plasma levels (14.3 ± 6.9 vs. 9.5 ± 4.7 ng/ml, *p* < 0.001). Solely sICAM-1 reduction persisted for 25 ± 5 h between the first and second TPE treatment, while the other investigated mediators increased to baseline levels. Substantial amounts of all measured mediators could be recovered from the removed plasma.

**Conclusions:**

TPE provides a persistent reduction in sICAM-1 levels and temporarily affects several adipokine and cytokine plasma levels. Our findings are of importance not only for the interpretation of blood levels of cytokines in patients undergoing TPE but provide solid evidence that TPE markedly decreases sICAM-1.

## Background

Therapeutic plasma exchange (TPE) is an extracorporeal treatment modality separating and removing blood plasma and replacing it with a protein containing fluid such as albumin [1]. It is performed in an increasing number of mainly immunologic disorders to remove substances with a high molecular weight such as antibodies, antibody-antigen complexes, and paraproteins [2]. In addition, due to the unselective removal of plasma, other plasma components like inflammatory mediators get eliminated as well. This may play a role in inflammatory states, as for example sepsis with multi-organ failure, where TPE has been employed [3–5]. So far, data regarding the removal of inflammatory mediators during TPE are scarce. This is especially true for adipokines, which have lately been found to mediate inflammatory processes. The adipose tissue, the origin of these substances, is described as the body’s largest endocrine active organ, which contributes to a chronic low-grade inflammatory state in obese patients [6]. In this regard, the adipokine leptin is known to influence mammals’ food intake and energy balance as well as inflammatory processes after stimulated by cytokines or lipopolysaccharides [7–9]. Intercellular Adhesion Molecule 1 (ICAM-1) orchestrates the migration of inflammatory cells [10]. sICAM-1, the soluble form of ICAM-1, has been shown to be elevated and of pathophysiological importance in immunologic disorders as vasculitis [11], a condition for which TPE is used on a regular basis [12]. Besides their key role in regulating inflammatory processes, adipokines and cytokines are also biomarkers in numerous disorders, where their plasma level is related to the disease activity like in systemic lupus erythematodes [13, 14]. In general, sICAM-1 is viewed as a biomarker for endothelial activation [15].

The aim of this study was to investigate the effect of TPE on inflammatory markers / adipokines, to quantify their removal, and to assess their rebound after the treatment.

## Methods

The study was approved by the local Ethics Committee of Hannover Medical School, Germany protocol # 5343. All patients gave written informed consent before enrolment into the study. We started the study with 21 Caucasian patients (10 females and 11 males with a mean age of 51.6 ± 13.5 years and a BMI of 25.1 ± 5.0 kg/m^2^) with indication for TPE due to various diseases including humoral rejection after solid organ transplantation, Guillain-Barré syndrome, monoclonal gammopathy, multiple sclerosis, rapid progressive glomerulonephritis, polyneuritis, microscopic polyangitis, and cryoglobulinemia. Further patients’ characteristics and details of the procedure are described elsewhere [16].

Every patient received two consecutive TPE sessions during the study. Plasma exchange therapy was performed using either the Spectra Optia® (TerumoBCT Inc., USA) or the Octo Nova® (DIAMED Medizintechnik GmbH, Germany) apheresis system. Anticoagulation was applied either by heparin or citrate. The prescribed dose of exchange volume of every TPE treatment was 1.1-times the individual calculated total plasma volume, using the Nadler-Allen equation.

A substitute fluid with 5 % albumin concentration was used in every treatment. Blood samples for measurement of different adipokines / obesity markers as resistin (12.5 kDa), leptin (16 kDa), sICAM-1 (80–110 kDa), soluble CD40 ligand (sCD40L, 39 kDa), monocyte chemoattractant protein-1 (MCP-1, 13 kDa), soluble tumor necrosis factor receptor (sTNF-R, 60 kDa), and routine chemistry were drawn before (pre-TPE) and at the end (post-TPE) of the first and second TPE session. Samples at the end of each TPE treatment were collected before the rinse back of the blood. Additionally, plasma samples from the waste bags were drawn after each treatment. Blood samples were immediately cooled on ice, centrifuged at 1500 g, and 4 °C for 10 min. Plasma samples were stored in 1 ml aliquots at −80 °C until further use.

### Analysis of plasma adipokines and cytokines

Leptin, resistin, soluble CD40 ligand (sCD40L), sICAM-1, soluble tumor necrosis factor receptor (sTNF-R), and monocyte chemoattractant protein 1 (MCP-1) were analysed using the eBioscience® FlowCytomix™ Human Obesity 9plex Kit (Bender MedSystems GmbH, Austria) following the manufacturer’s instructions. A standard protein dilution and human plasma samples were incubated with a Bead and Conjugate Mixture for two hours. After washing with assay buffer, a Streptavidin-PE Solution was added and incubated for one hour. Subsequently, samples and standard protein dilutions were washed twice, re-suspended in assay buffer, and analysed by flow cytometry using LSR Fortessa (BD Biosciences, San Diego, USA) with FlowCytomix Pro Software (Bender MedSystems GmbH, Vienna, Austria).

### Statistical analysis

We used GraphPad Prism 6 (GraphPad Software, Inc., La Jolla, USA) for statistical analysis. Pre- and post-TPE levels of all treatments were compared suing the student T-test. This analysis was conducted in the whole study population as well as woman and men separately, the significance level was set at *p* < 0.05.

## Results

All enrolled patients completed the two study TPE treatments. The exchanged plasma volume over all treatment sessions was 3570 ± 589 ml, which was 1.1 ± 0.1-times the individual calculated plasma volume. The average time period between the first and second therapy session was 25 ± 5 h.

### sICAM-1

**Fig. 1:**
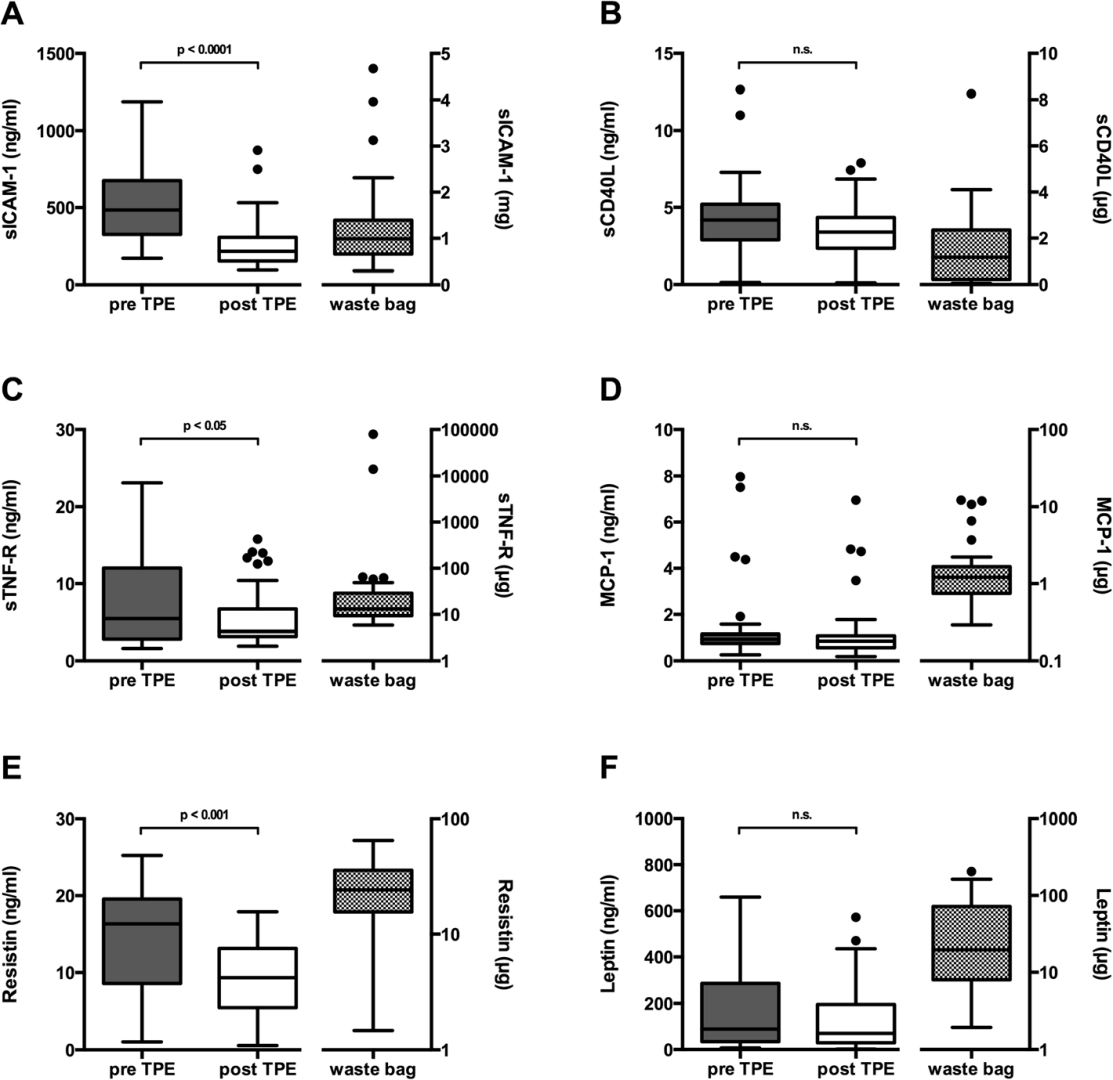
Pre- and post-TPE plasma levels (left y-axis) of adipokines and cytokines levels summarizing all treatments. Total amount of the respective compounds in the waste bag is shown on the right y-axis. Removal of each investigated molecule is visualized in separated panels (**a**: sICAM-1, **b**: sCD40L, **c**: sTNF-R, **d**: MCP-1, **e**: Resistin, **f**: Leptin)

**Fig. 2:**
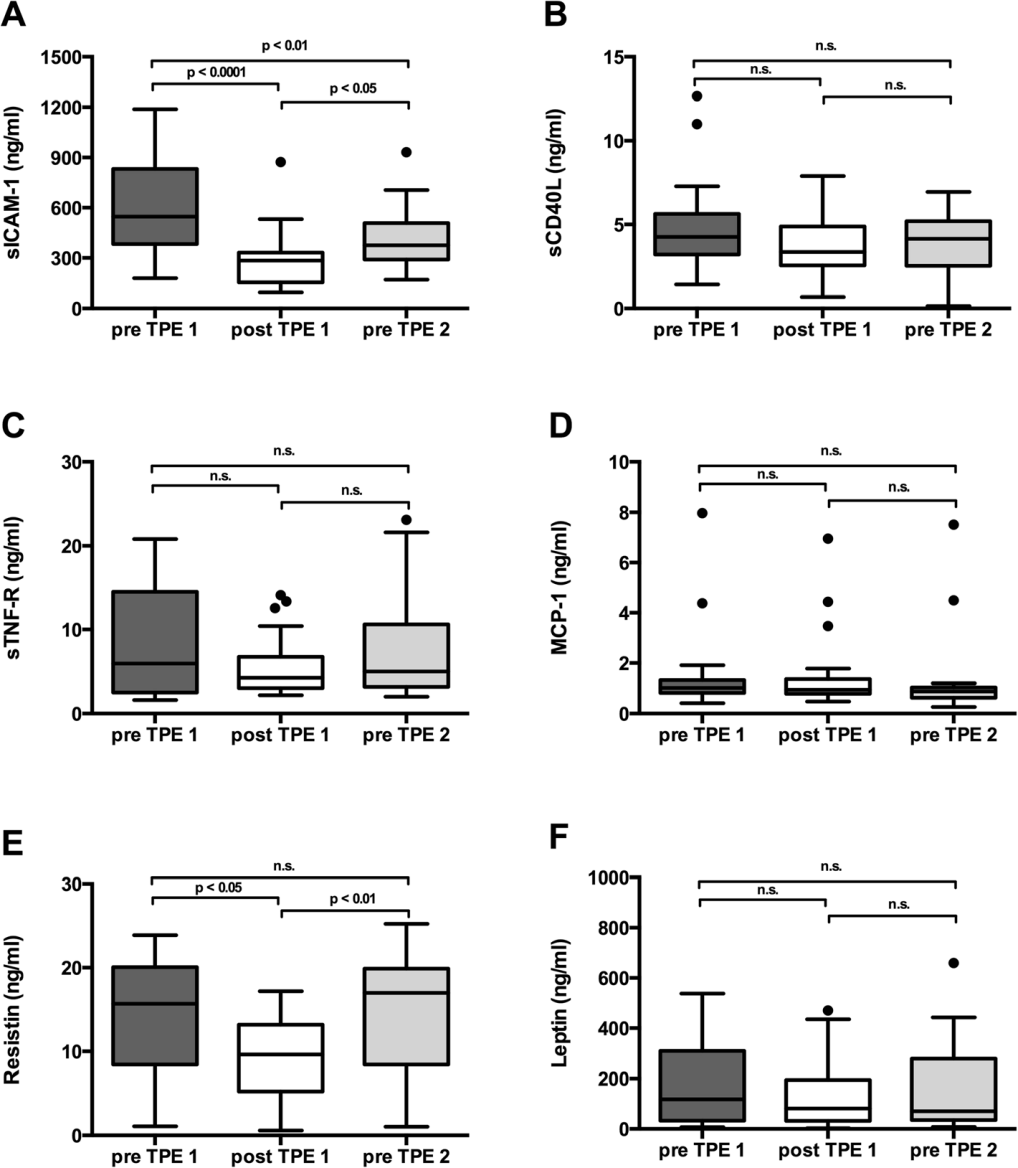
Time course of adipokine and cytokine plasma levels. Time between end of 1^st^ treatment (post TPE 1) and beginning of 2^nd^ treatment (pre-TPE 2) was 25 ± 5 h. Plasma levels of each investigated molecule are visualized in separated panels (**a**: sICAM-1, **b**: sCD40L, **c**: sTNF-R, **d**: MCP-1, **e**: Resistin, **f**: Leptin)

Evaluating all TPE sessions (1^st^ and 2^nd^) plasma levels of sICAM-1 were reduced by ~ 50 % (from 517 ± 246 pre-TPE to 260 ± 159 ng/ml post TPE, *p* < 0.0001). Separate analysis of the 1^st^ TPE session showed a decrease of sICAM-1 from 615 ± 261 to 291 ± 173 ng/ml, *p* < 0.0001. Despite a rebound from 291 ± 173 after the 1^st^ TPE to 418 ± 189 ng/ml (*p* < 0.05) before the 2^nd^ these levels were still lower than the ones before the 1^st^ treatment (418 ± 189 ng/ml vs. 615 ± 261, *p* < 0.01), suggesting a prolonged reduction effect of TPE on sICAM-1. A total amount of 1.3 ± 0.9 mg sICAM-1 per treatment was recovered from the total removed collected plasma (Figs. 1a and 2a).

### sCD40L

There was neither a significant decrease in soluble CD40 Ligand plasma levels comparing all pre-TPE and post-TPE levels (4.4 ± 2.3 vs. 3.4 ± 1.7 ng/ml, n.s.) nor analysing the effect of the 1^st^ TPE alone (4.8 ± 2.7 vs. 3.6 ± 1.8 ng/ml, n.s.). Furthermore, no significant differences of sCD40L neither in pre TPE plasma levels between the two treatment sessions (pre-TPE 1 vs. pre-TPE 2: 4.8 ± 2.7 vs. 3.9 ± 1.7 ng/ml, n.s.) nor from the end of the 1^st^ to the beginning of the 2^nd^ treatment (3.6 ± 1.8 vs. 3.9 ± 1.7 ng/ml, n.s.) could be detected. We found a total amount of 1.6 ± 1.8 μg sCD40L in the collected removed plasma. (Figs. 1b and 2b).

### sTNF-R

There was a significant decrease in soluble TNF-R comparing all pre-TPE to post-TPE plasma levels (8.1 ± 6.4 vs. 5.7 ± 3.9 ng/ml, *p* < 0.05). We found a total amount of 21.3 ± 18.6 μg sTNF-R in the collected removed plasma. However, there was no significant decrease in TNF-R plasma levels comparing pre to the post-TPE levels of the 1^st^ TPE treatment alone (8.3 ± 6.7 vs. 5.6 ± 3.8 ng/ml, n.s.). There was also no significant difference in pre-TPE plasma levels of sTNF-R between the two treatment sessions (8.3 ± 6.7 vs. 7.8 ± 6.3 ng/ml, n.s.) nor a sTNF-R increase from the end of the 1^st^ to the beginning of the 2^nd^ treatment (5.6 ± 3.8 vs. 7.8 ± 6.3 ng/ml, n.s.) (Figs. 1c and 2c).

### MCP-1

There was neither a significant decrease in MCP-1 comparing all pre-TPE to post-TPE plasma levels (1.4 ± 1.7 vs. 1.2 ± 1.3 ng/ml, n.s.) nor looking at the 1^st^ TPE alone (1.5 ± 1.7 vs. 1.3 ± 1.4 ng/ml, n.s.). Despite the lack of effect of TPE on plasma levels we found a total amount of 2.1 ± 2.9 μg MCP-1 in the exchanged plasma. (Figs. 1d and 2d).

### Resistin

Resistin was significantly reduced during TPE comparing all pre- and post-TPE sessions (14.3 ± 6.9 vs. 9.5 ± 4.7 ng/ml, *p* < 0.001) as well as looking at the first TPE alone (14.2 ± 6.9 vs. 9.4 ± 4.5 ng/ml, *p* < 0.05). A total amount of 26.5 ± 16.3 μg resistin was detected in the removed plasma. There was a significant rebound from the end of the 1^st^ to the beginning of the 2^nd^ treatment (9.4 ± 4.5 vs. 14.5 ± 7.1 ng/ml, *p* < 0.01). However, the resistin plasma levels prior to the first and second treatment did not differ significantly (14.2 ± 6.9 vs. 14.5 ± 7.1 ng/ml, n.s.) (Figs. 1e and 2e).

### Leptin

There was neither a significant reduction of leptin plasma by TPE comparing all pre-TPE and post-TPE levels (172.8 ± 169.4 vs. 122.4 ± 134.2 ng/ml, n.s.) nor analysing the effect of the 1^st^ TPE separately (178.5 ± 159.4 vs. 130.5 ± 135.5 ng/ml, n.s.). We found a total amount of 45.2 ± 49.2 μg leptin in the total removed plasma. No significant increase in leptin plasma levels between the end of the 1^st^ treatment and the start of the 2^nd^ TPE (130.5 ± 135.5 vs. 167.1 ± 181.8 ng/ml, n.s.). (Figs. 1f and 2f).

### Gender effects

Interestingly, in a separate analysis on the effect of gender the female patients revealed a resistin reduction during the 1^st^ TPE treatment (pre-TPE 1_women_ vs. post-TPE 1_women_: 17.1 ± 5.7 vs. 11.8 ± 3.8 ng/ml, *p* < 0.05) as well as resistin rebound (post-TPE 1_women_ vs. pre-TPE 2_women_: 11.8 ± 3.8 vs. 17.3 ± 5.8 ng/ml, *p* < 0.05) while men did not. Plasma resistin levels were also significantly higher in women as compared to the male patients before and after TPE (pre-TPE_women_ vs. pre-TPE_men_: 17.2 ± 5.6 vs. 11.2 ± 6.9 ng/ml, *p* < 0.01; post-TPE_women_ vs. post-TPE_men_: 11.8 ± 3.9 vs. 7.1 ± 4.3 ng/ml, *p* < 0.001).

## Discussion

This study shows for the first time that sICAM-1, sTNF-R, and resistin plasma levels are significantly reduced by TPE treatment and quantifies the amount of these substances in the total collected removed plasma. While the effect for sICAM-1 persisted for 25 ± 5 h, all other investigated markers increased back to baseline in that period of time. These results can also be found in a subgroup analysis, evaluating patients with BMI > 25 kg/m^2^ and with a BMI < 25 kg/m^2^ seperatey. There is a gender specific effect of TPE on resistin plasma levels.

There are several aspects to consider in the interpretation of our results. It seems unlike that, the difference in prolonged serum level decrease between the investigated substances is due their difference in molecular size. All investigated substances, which range from 12.5 to 89 kDa are clearly below the molecular cut-off of the TPE, i.e.1000 kDa.. Different serum half-life times and different volumes of distribution seem more likely to play a role in the different effects of TPE in the examined markers. This is best epitomized by leptin, which it is not significantly altered by TPE. In the human body it has a very short half-life of 25 min [17]. The half-life of sICAM-1, the molecule which was markedly and prolonged lowered by TPE, is only ill defined as it is highly variable and changes with the underlying disease. However, induced sICAM-1 release in human epithelial cells needs up to 24 h to achieve a steady state concentration [18], therefore the sICAM-1 serum dynamic is presumably slower than the one of leptin. Adipokine serum levels, except resistin, were generally higher in our patients than in healthy controls described in the literature.

While the effect of TPE on immunoglobulins and immune-complexes [19] or even cardiac markers like troponin and BNP [16] has been shown, data on the effect of other pathophysiologically important mediators are scarce. The most interesting compound in our analysis was sICAM-1. It increases in different inflammatory diseases like vasculitis or psoriasis arthritis [20]. It is believed that the soluble form of ICAM-1 has an active immunologic role due to its interaction with lymphocyte function-associated antigen (LFA-1) [11]. Compared to healthy subjects reported in the literature, the observed plasma concentrations of our patients was markedly elevated. This is in line with previous studies in patients with inflammatory diseases [21]. In ANCA-positive renal vasculitis Tesar et al. proved a significant decrease of sICAM-1 in the TPE treatment period [22]. In our study, we see a marked effect on TPE on sICAM in all patients including those with antibody-mediated rejection after renal transplantation. This might be of clinical importance as sICAM-1 levels significantly correlate with mortality in renal transplant recipients [23]. To our knowledge this is the first time both, a significant decrease of sICAM-1 levels in the patient’s blood after TPE and the eliminated whole amount of sICAM-1 in the waste bag, are shown.

Soluble TNF-R is important in inflammatory conditions like spondyloarthritis and is associated with delirium in critically ill patients [24, 25]. sTNF-R levels are increased in our patient group compared with the general population [26]. In the present study we observed a significant decrease in sTNF-R levels of 30 % during TPE treatment. This finding is remarkable, as even extracorporeal liver support systems as MARS™ (Molecular Adsorbent Recirculating System) or Prometheus™ were not able to lower sTNF-R levels [27]. However, the sTNF-R levels did not differ in pre TPE levels between the 1^st^ and the 2^nd^ treatment suggesting a short lived effect on plasma levels, which does not exclude a potential clinical benefit in diseases like spondyloarthritis.

The adipokine leptin could not be lowered by TPE. The role of leptin in inflammatory diseases is controversial. It might be a marker of disease activity in Behçet’s syndrome and is elevated in patients with systemic lupus erythematodes [28, 29]. Leptin also seems to decrease in patients with active ANCA associated vasculitis [30]. It is possible that leptin plays an important role in several inflammatory conditions. Yet, we found no significant decrease in leptin plasma levels in our study.

The role of resistin in inflammation is also a matter of debate. While high resistin levels are associated with rheumatoid arthritis, coronary heart disease and insulin resistance, a low resistin plasma level may be correlated with a high hospitalization rate in dialysis patients [31, 32]. Interestingly, the women in our study had higher resistin plasma levels as compared to the male patients. Furthermore, resistin plasma levels decreased significantly in women during TPE. Gender specific differences in resistin levels have been described since the introduction of the molecule [33]. Steppan et al. speculate in a later review that gender differences may be due to differences in body fat distribution or hormone levels [34]. Hormone influence on resistin serum levels seems likely as they are approximately two times higher during premenopause compared to peri- or postmenopause [35]. However, estradiol administration or ovariectomy did not alter resistin serum levels in women [36]. Resistin serum levels seem to be independent from patients’ bodyweight and may be a possible risk factor for insulin resistance [37]. In addition, resistin may play a role in breast and endometrial cancer [38]. However, gender specific differences in resistin serum levels are not fully understood and cannot be further clarified by this study, as it was not designed to do so.

Excessive body fat mass in obese patients leads to elevated circulating adipokine levels especially of leptin, resistin, and TNF-α [39, 40]. We performed a subgroup analysis, where we divided our patients into a group with BMI <25 (7 women, 3 men) and >25 (4 women, 7 men). Interestingly, post TPE serum levels for resistin were significantly higher in the lean patient group, while pre TPE serum levels had a similar trend (pre TPE serum levels BMI <25 vs. BMI >25 16 ng/ml ± 1.6 vs. 11.9 ± 1.3 ng/ml, *p* = 0.06, post TPE serum levels BMI <25 vs. BMI >25: 10.7 ± 1.1 ng/ml vs. 7.9 ± 0.9 ng/ml, *p* < 0.05). Therefore, one could postulate, that the gender specific effect on resistin serum concentrations seems to overcome influences of body mass. However, due to the small patient number of this study, subgroup analyses could be misleading. Leptin, one of the central adipocyte-derived hormones, has a major influence on metabolism, immune functions [41], and has often been associated with cell proliferation and cancer development. A reduction of these mediators after therapeutic plasma exchange could accomplish positive effects in case of an obesity-related increase of adipokine levels. Furthermore, we demonstrated that altered levels of several adipokines (e.g. resistin and leptin) can decrease the glomerular filtration rate and increase albuminuria [42].

We wish to acknowledge several limitations of our study. Firstly, we only included patients receiving TPE with albumin as replacement fluid as fresh frozen plasma would have interfered with the analysis of the biomarkers. Therefore, our results are valid for patients treated with TPE and albumin as an exchange fluid. Furthermore, underlying illnesses necessitating TPE and other patient characteristics spanned a variety of conditions. Different morbidities may affect the kinetics of the measured marker, as well as differences in the timespan between the treatment sessions. This could lead to an over- or underestimation of the effects of TPE. Additionally, the patient number of our study is rather small and we did not include a follow up period, so clinical relevance of this date cannot be determined.

## Conclusion

In summary, the present study shows a substance- and time-specific removal of various adipokines by TPE. sICAM-1 is the only investigated substance, which shows a prolonged reduction in the patients’ serum levels by TPE. Therefore, sICAM-1 plasma levels should be interpreted with caution in patients undergoing TPE. Thus, apart from the removal of autoantibodies and proteins levels of adipocyte tissue derived hormones are altered by TPE. Depending on the specific adipokine, this side-effect of TPE may be (dis)advantageous for the treatment of the underlying disease.

## Consent

All patients gave written informed consent before enrolment into the study. A copy of the written consent is available for review by the Series Editor of this journal.

## Abbreviations

LFA-1: lymphocyte function-associated antigen
MARS™: Molecular Adsorbent Recirculating System
MCP-1: monocyte chemoattractant protein-1
ICAM-1: Intercellular Adhesion Molecule 1
sCD40L: soluble CD40 ligand
sTNF-R: soluble TNF-R
TPE: Therapeutic plasma exchange
